# Long-term outcomes of patients with end-stage kidney disease due to membranous nephropathy: A cohort study using the Australia and New Zealand Dialysis and Transplant Registry

**DOI:** 10.1371/journal.pone.0221531

**Published:** 2019-08-23

**Authors:** Wen-ling Yang, Bhadran Bose, Lei Zhang, Megan Mcstea, Yeoungjee Cho, Magid Fahim, Carmel M. Hawley, Elaine M. Pascoe, David W. Johnson

**Affiliations:** 1 Department of Nephrology, Peking University Third Hospital, Beijing, China; 2 Division of Nephrology, Princess Alexandra Hospital, Brisbane, Australia; 3 Australia and New Zealand Dialysis and Transplant (ANZDATA) Registry, Adelaide, Australia; 4 Australasian Kidney Trials Network, The University of Queensland, Queensland, Australia; 5 Department of Nephrology, Nepean Hospital, Kingswood, Australia; 6 Department of Nephrology, Guangdong Provincial Hospital of Chinese Medicine, Guangzhou, China; 7 Translational Research Institute, Brisbane, Australia; University of Utah School of Medicine, UNITED STATES

## Abstract

**Background:**

Clinical outcomes of patients with end-stage kidney disease (ESKD) secondary to membranous nephropathy (MN) have not been well described. This study aimed to evaluate patient and/or allograft outcomes of dialysis or kidney transplantation in patients with ESKD secondary to MN.

**Material and methods:**

All adult patients with ESKD commencing renal replacement therapy in Australia and New Zealand from January 1998 to December 2010 were extracted retrospectively from ANZDATA registry on 31^st^ December 2013. Outcomes of MN were compared to other causes of ESKD. In a secondary analysis, outcomes of MN were compared to all patients with ESKD due to other forms of glomerulonephritis.

**Results:**

Of 32,788 included patients, 417 (1.3%) had MN. Compared to other causes of ESKD, MN experienced lower mortality on dialysis (adjusted hazard ratio [aHR] 0.79, 95% CI 0.68–0.92, p = 0.002) and following kidney transplantation (aHR 0.57, 95% CI 0.33–0.97, p = 0.04), had a higher risk of death-censored kidney allograft failure (aHR 1.55, 95% CI: 1.00–2.41, p = 0.05) but comparable risk of overall kidney allograft failure (aHR 1.35, 95% CI 0.91–2.01, p = 0.13). Similar results were obtained using competing-risk regression analyses. MN patients were significantly more likely to receive a kidney transplant (aHR 1.38, 95% CI 1.16–1.63, p<0.001) and to experience primary kidney disease recurrence in the allograft (aHR 4.92, 95% CI 3.02–8.01, p<0.001). Compared to other forms of glomerulonephritis, MN experienced comparable dialysis and transplant patient survival, but higher rates of kidney transplantation, primary renal disease recurrence and death-censored allograft failure.

**Conclusion:**

MN was associated with superior survival on dialysis and following kidney transplantation compared to patients with other causes of ESKD, and comparable patient survival compared to patients with other forms of glomerulonephritis. However, patients with MN exhibited a higher rate of death-censored allograft loss as a result of primary kidney disease recurrence.

## Introduction

Membranous nephropathy (MN) is one of the most common types of glomerular disease and accounts for up to 20–40% of nephrotic syndrome in adults [1–3]. It is observed across all ethnicities and ages. Approximately, 70%-80% of cases of MN in adults are idiopathic and the rest are attributed to various secondary causes, including infections, autoimmune disorders and malignancy [2–5]. Although it is reported to be one of the most common forms of primary glomerulonephritis (GN) to result in end-stage kidney disease (ESKD) and carries very high risk of recurrence following kidney transplantation (up to 40%) [5, 6], the clinical outcomes of patients with MN receiving renal replacement therapy (RRT) have not been well described [4, 5, 7, 8].

The aim of this study was to evaluate patient and/or allograft outcomes of dialysis or kidney transplantation in patients with ESKD secondary to MN, using data from the Australia and New Zealand Dialysis and Transplant (ANZDATA) Registry.

## Materials and methods

### Study population

This retrospective cohort study used the records of adult patients who commenced RRT from January 1998 to December 2010 which were extracted from the ANZDATA Registry on 31^st^ December 2013. And only those with enough data as listed in the following covariates part were analyzed. Patients were initially categorized into two groups according to the cause of ESKD: MN and all other causes. MN was diagnosed by kidney biopsy and clinical criteria [5, 9, 10]. In a secondary analysis, all patients with ESKD due to glomerulonephritis (GN) were separately categorized into MN and other GN groups. The study adhered to the Declaration of Helsinki, Istanbul. Research on ANZDATA Registry data was approved by the review board of Princess Alexandra Hospital (HREC/03/QPAH/32) and ANZDATA executive. The data were analyzed anonymously, so the written consent was not obtained.

The outcomes examined for dialysis patients were patient survival, dialysis-independent recovery of kidney function, and probability of receiving a kidney transplant. Dialysis patient survival analyses included only those patients who received dialysis as their first RRT. Recovery of kidney function was defined as the date of the last dialysis treatment without requirement for further RRT. For kidney transplant patients, outcomes included primary kidney disease recurrence in the allograft, allograft survival and patient survival. Onset of allograft failure was defined as the date of commencement of dialysis after transplantation. For patients who received more than one transplant, only survival of the first allograft was analyzed.

### Statistical analysis

Patient characteristics were expressed as frequencies and percentages for categorical variables, mean ± standard deviation (SD) for continuous normally distributed variables, and median (interquartile range [IQR]) for continuous variables that were not normally distributed. Characteristics of ESKD secondary to MN were compared to those of ESKD due to other causes using Pearson’s chi-square for categorical variables, two-tailed unpaired t-tests or Mann-Whitney tests for continuous variables, depending on data distribution.

Time-to-event outcomes were analyzed using multivariable Cox proportional hazards regression. Patient survival on dialysis was censored for kidney function recovery, loss to follow-up, kidney transplantation and end of study. Patient survival for kidney transplant recipients was censored for allograft failure, loss to follow-up and end of study. Death-censored kidney allograft survival was censored for death, loss to follow-up and end of study. Covariates included in the Cox models were age, gender, racial origin, dialysis era or transplant era, first RRT treatment, smoking status, body mass index (BMI), late referral (referral to a renal unit within 3 months of RRT commencement), and comorbidities recorded at registry entry (including cerebrovascular disease [CVD], coronary artery disease [CAD], diabetes mellitus [DM], peripheral vascular disease [PVD] and chronic lung disease). Donor type and the number of previous kidney transplants were additional covariates examined for the survival analysis of transplant patients. Proportional hazards assumptions were checked by formal hypothesis testing and graphically display of Schoenfeld residuals. In view of the possibility of informative censoring due to differential competing-risk rates between patients with and without MN, sensitivity analyses were performed using a competing-risk approach: kidney transplantation and kidney function recovery were competing events in the survival analysis for patients on dialysis; kidney transplantation was the competing risk for dialysis-independent kidney function recovery; patient death was the competing risk in kidney allograft survival and allograft failure was the competing risk for patient survival after kidney transplantation. Statistical analysis was performed using Stata version 14.0 (StataCorp., College Station, TX). P values <0.05 were considered to be statistically significant.

## Results

### Patient characteristics

**Fig 1:**
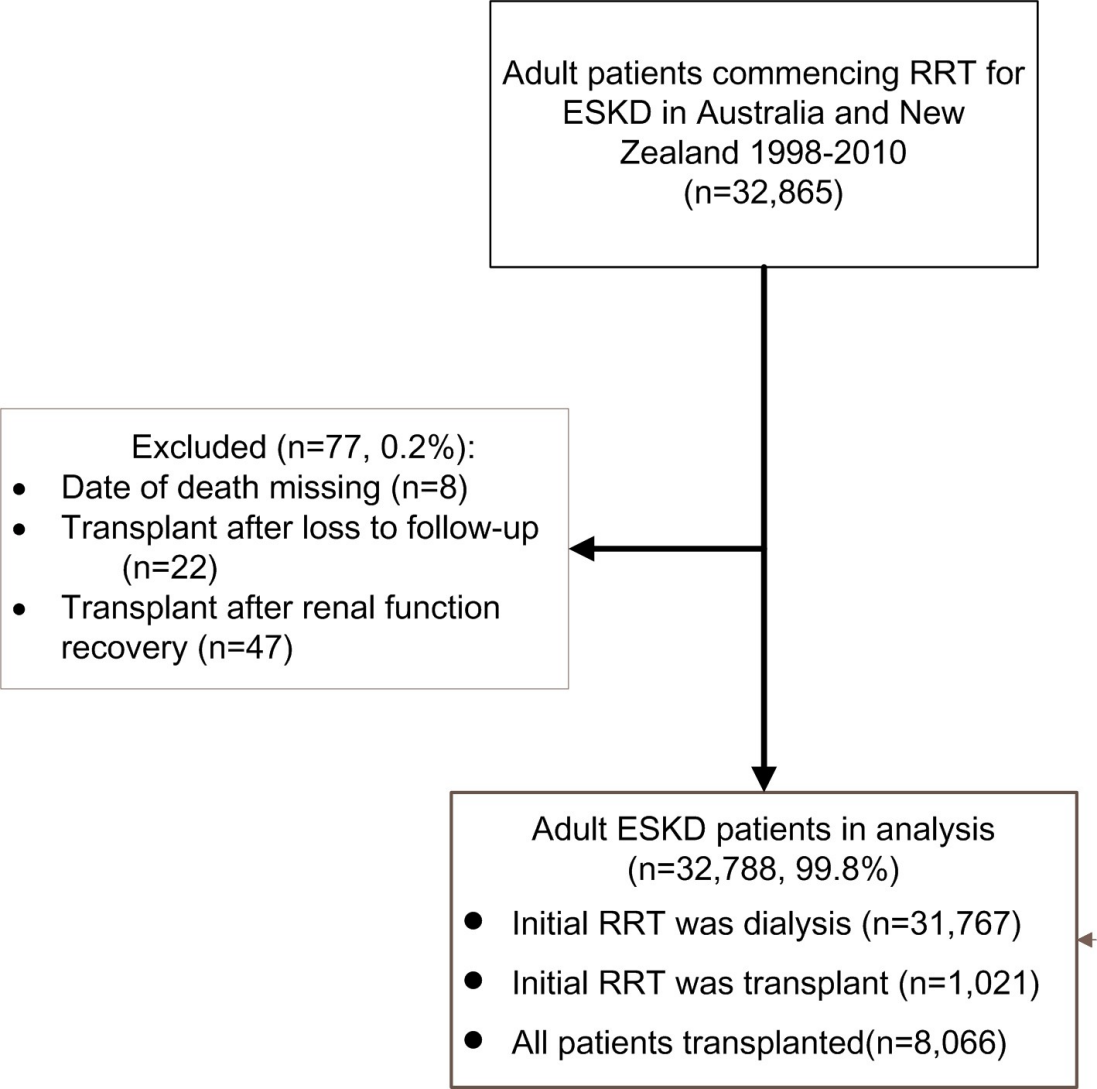
Flow chart. Abbreviations: RRT,Renal replacement therapy. ESKD, End-stage kidney diseases.

Between 1^st^ January 1998 and 31^st^ December 2010, 32,865 adult patients started RRT for ESKD in Australia and New Zealand. Of these, 77 patients were excluded due to data gaps (Fig 1). There were 417 (1.3%) patients with ESKD secondary to MN, of whom 388 (1.2%) underwent dialysis as their first RRT with a median follow-up of 3.3 years [interquartile range [IQR] 1.6–5.6]. Compared to patients with ESKD from other causes (n = 31,379), MN patients were disproportionately male (74% vs 60%), Caucasian (87% vs 73%), and significantly less likely to suffer from comorbidities (DM [16% vs 43%], CAD [35% vs 41%], PVD [17% vs 27%]) and to have a late referral (18% vs 24%) (Table 1).

**Table 1:** Characteristics of ESKD patients whose first RRT was dialysis in Australia and New Zealand.

Characteristics	Membranous nephropathy (n = 388)	Other cause of ESKD (n = 31,379)	P value
**Age (years)**	61[51–70]	62[50–72]	0.33
18–29	12(3%)	1,328(4%)	0.04
30–39	27(7%)	2,194(7%)	
40–49	45(12%)	4,120(13%)	
50–59	98(25%)	6,316(20%)	
60–69	105(27%)	7,587(24%)	
≥70	101(26%)	9,834(32%)	
**Male**	288(74%)	18,755(60%)	<0.001
**Racial origin**			<0.001
White	337(87%)	22,847(73%)	
ATSI	11(3%)	2,454(8%)	
MPI	19(5%)	3,365(10%)	
Asian	17(4%)	2,159(7%)	
Other	4(1%)	554(2%)	
**RRT era**			0.23
1998–2002	142(37%)	10,414(33%)	
2003–2007	156(40%)	12,647(40%)	
2008–2012	90(23%)	8,318(27%)	
**Smoking status at RRT entry**			0.15
Current	60(15%)	4,225(13%)	
Former	170(44%)	12,843(41%)	
Never	158(41%)	14,285(46%)	
**Diabetes mellitus**	63(16%)	13,574(43%)	<0.001
**Chronic lung disease**	70(18%)	5,193(17%)	0.43
**Coronary artery disease**	134(35%)	12,963(41%)	0.01
**Peripheral vascular disease**	67(17%)	8,372(27%)	<0.001
**Cerebrovascular disease**	48(12%)	4,844(15%)	0.10
**BMI (kg/m**^**2**^**)**	26.0[22.8–29.4]	26.4[23.1–30.8]	0.06
Underweight (<18.5)	13(3%)	1,097(4%)	0.08
Normal weight (18.5–24)	152(39%)	11,165(36%)	
Overweight (25–29)	136(35%)	10,107(32%)	
Obese (≥30)	87(23%)	8,850(28%)	
**Late referral**	70(18%)	7,625(24%)	0.004
**First RRT**			0.08
Hemodialysis	271(70%)	23,137(74%)	
Peritoneal dialysis	117(30%)	8,242(26%)	
**Follow-up years**	3.3[1.6–5.6]	3.2[1.4–5.3]	0.14
**Native kidney biopsy**	388(100%)	9,371(30%)	<0.001

Abbreviations: ESKD, End-stage kidney disease; RRT, Renal replacement therapy;ATSI, Aboriginal and Torres Strait Islander; MPI, Maori and Pacific Islander; BMI, body mass index

When compared to patients with other forms of GN (n = 7,409), MN patients were more likely to be older (median age: 61 vs 55 years old), male (74% vs 63%) and Caucasian (87% vs 75%) with a greater comorbidity burden (DM [16% vs 13%], CAD [35% vs 25%], PVD [17% vs 11%], CVD [12% vs 9%], Chronic lung disease [18% vs 14%]) and likelihood of choosing peritoneal dialysis (PD) as the first RRT (30% vs 26%). Also, they were less likely to be referred late to renal units (18% vs 24%) (S1 Table).

### Patient survival on dialysis

**Fig 2:**
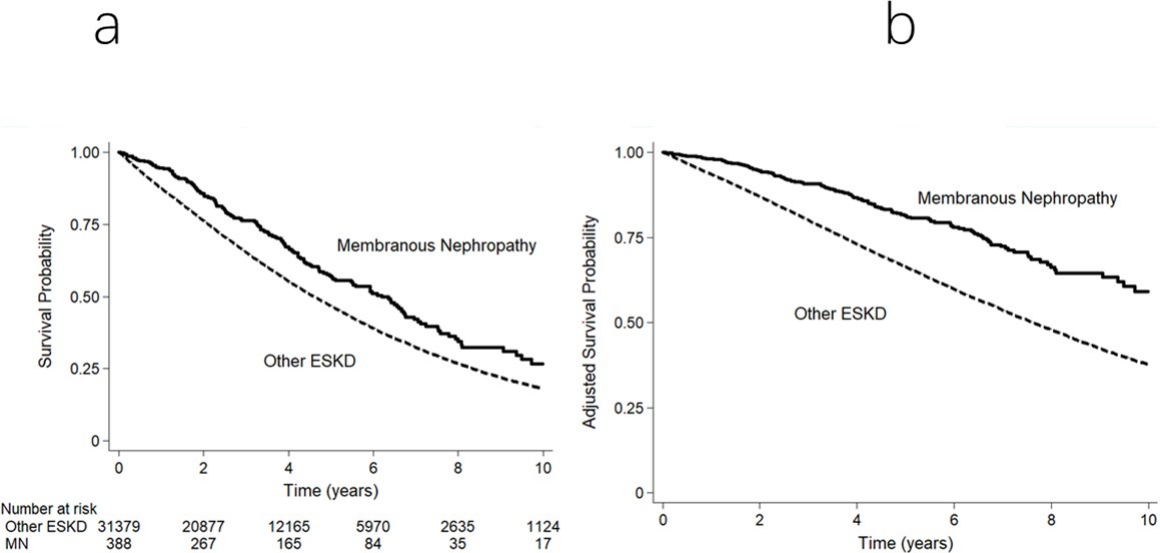
Survival curves for ESKD secondary to membranous nephropathy and other causes in patients commencing dialysis in Australia and New Zealand. (a) Unadjusted Kaplan–Meier survival curve. (b) Survival curve adjusted for demographic and comorbidity indices. The difference between the 2 groups was significant (unadjusted p < 0.001; adjusted p = 0.002).

There were 169 deaths (44%) amongst MN patients (132 per 1000 patient-years) on dialysis compared to 17,947 (57%) patients with ESKD from other causes (212 per 1000 patient-years; p<0.001). There was no difference in the causes of death between MN and other causes of ESKD (S2 Table). Respective adjusted patient survival rates at 1, 5, and 10-years were 98%, 81% and 58% for MN and 93%, 65% and 36% for other ESKD causes (Fig 2). ESKD from MN was associated with a significantly lower mortality risk (adjusted hazard ratio [aHR] 0.79, 95% CI: 0.68–0.92, p = 0.002). Competing-risk survival analysis showed similar results (sub-hazard ratio [SHR] 0.74, 95% CI 0.64–0.86, p< 0.001).

When patients with MN were compared to patients with other forms of GN, comparable survival was observed in both Cox regression analysis (aHR 0.94, 95% CI 0.80–1.10, p = 0.42) and competing risk analysis (SHR 0.87, 95% CI 0.74–1.03, p = 0.10).

### Recovery of dialysis-independent kidney function

Recovery of kidney function occurred in 10 MN patients (3%), 523 patients with other causes of ESKD (2%, p = 0.17) and 134 patients with other Glomerulonephritis (2%, p = 0.27). MN was not associated with kidney function recovery in either Cox regression analysis (aHR 1.52, 95% CI 0.81–2.84, p = 0.19; S1 Fig) or competing-risk analysis (SHR 1.60, 95% CI 0.85–3.00, p = 0.14; S2 Fig). Similar results were obtained when comparing MN with other GN patients (aHR 1.50, 95% CI 0.78–2.86, p = 0.22; SHR 1.56, 95% CI 0.81–3.00, p = 0.18).

### Likelihood of kidney transplantation

For those who commenced RRT on dialysis, kidney transplantation occurred in 138 (36%) patients with MN and 6,907 (22%) patients with other ESKD causes during the study period (p<0.001). There was no significant difference in the median time from dialysis commencement to first kidney transplant (2.2 years vs 2.3 years, p = 0.82) compared to patients with ESKD from other causes. However, MN patients were significantly more likely to undergo kidney transplantation compared to patients with ESKD from other causes (SHR 1.48, 95% CI 1.23–1.77, p<0.001, with the cumulative hazard curves listed in S3 Fig) and those with other forms of GN (SHR 1.21, 95% CI 1.02–1.44, p = 0.03).

### Primary kidney disease recurrence in kidney transplants

Characteristics of patients with ESKD due to MN who underwent their first kidney allograft compared to all other ESKD patients or compared to other types of GN are listed in Table 2 and S3 Table, respectively. Nineteen MN patients (11.4%) experienced MN recurrence in their allografts after a median time of 3.6 (1.0–4.7) years, which progressed to allograft loss in nine patients (47%) after a median time of 3.0 (1.7–3.9) years from the diagnosis of recurrence (S4 Table). In contrast, primary kidney disease recurrence occurred in 171 (2.2%) patients with other forms of ESKD and 152 (4.5%) in patients with other forms of GN. Primary kidney disease recurrence was significantly more likely in patients with MN than in patients with other ESKD causes (aHR 4.92, 95% CI 3.02–8.01, p< 0.001) or other forms of GN (aHR 2.83, 95% CI 1.72–4.64, p<0.001).

**Table 2:** Characteristics of patients undergoing first kidney transplantation for ESKD in Australia and New Zealand.

Characteristics	Membranous nephropathy (n = 167)	Other ESKD (n = 7,899)	P value
**Recipient Age (years)**	53[40–61]	49[38–58]	0.01
18–29	13(8%)	838(11%)	0.047^a^
30–39	26(16%)	1,362(17%)	
40–49	34(20%)	1,906(24%)	
50–59	42(25%)	2,153(27%)	
60–69	45(27%)	1,467(19%)	
≥70	7(4%)	173(2%)	
**Male Recipient**	129(77%)	4,932(62%)	<0.001
**Recipient Race**			0.61^a^
White	143(86%)	6,391(81%)	
Asian	10(6%)	736(9%)	
ATSI	5(3%)	229(3%)	
MPI	6(3%)	330(4%)	
Other	3(2%)	213(3%)	
**Transplant era**			0.001
1998–2002	51(31%)	1,533(19%)	
2003–2007	45(27%)	2,687(34%)	
2008–2013	71(42%)	3,679(47%)	
**Smoking status at RRT entry**			0.37
Current	15(9%)	884(11%)	
Former	62(37%)	2,558(33%)	
Never	90(54%)	4,435(56%)	
**Diabetes mellitus**	9(5%)	1,471(19%)	<0.001
**Chronic lung disease**	16(10%)	383(5%)	0.01
**Coronary artery disease**	25(15%)	858(11%)	0.09
**Peripheral vascular disease**	9(5%)	507(6%)	0.59
**Cerebrovascular disease**	8(5%)	276(3%)	0.37
**BMI (kg/m**^**2**^**)**	25.9[22.8–28.6]	25.5[22.5–29.1]	0.74
Underweight (<18.5)	5(3%)	269(4%)	0.67
Normal weight (18.5–24)	69(41%)	3,343(42%)	
Overweight (25–29)	64(38%)	2,677(34%)	
Obese (≥30)	29(18%)	1,567(20%)	
**Late referral**	25(15%)	1,328(17%)	0.51
**First RRT**			0.07
Kidney transplantation	29(17%)	992(13%)	
Peritoneal dialysis	46(28%)	1,924(24%)	
Hemodialysis	92(55%)	4,983(63%)	
**EBV seropositive**	139(89%)	6,566(89%)	0.88
**CMV seropositive**	115(71%)	5,193(67%)	0.33
**Male donor**	84(51%)	4,022(52%)	0.80
**Male donor for female receipt**	18(11%)	1,602(20%)	0.002
**Deceased donor**	96(57%)	4,748(60%)	0.47
**Subsequent allografts**	5(3%)	224(2.84%)	0.82
2^nd^	5(3%)	219(2.773%)	
3^rd^ or more	0	5(0.063%)	
**Follow-up years**	5.6[3.2–10.4]	5.3[2.8–8.7]	0.07
**Native kidney biopsy**	167(100%)	3,730(47%)	<0.001

Abbreviations: ESKD, End-stage kidney diseases; ATSI, Aboriginal and Torres Strait Islander; MPI, Maori and Pacific Islander; RRT, Renal replacement therapy; BMI, body mass index; EBV, Epstein-Barr Virus; CMV, Cytomegalovirus

^a^ Fisher’s test result.

### Kidney transplant allograft survival

**Fig 3:**
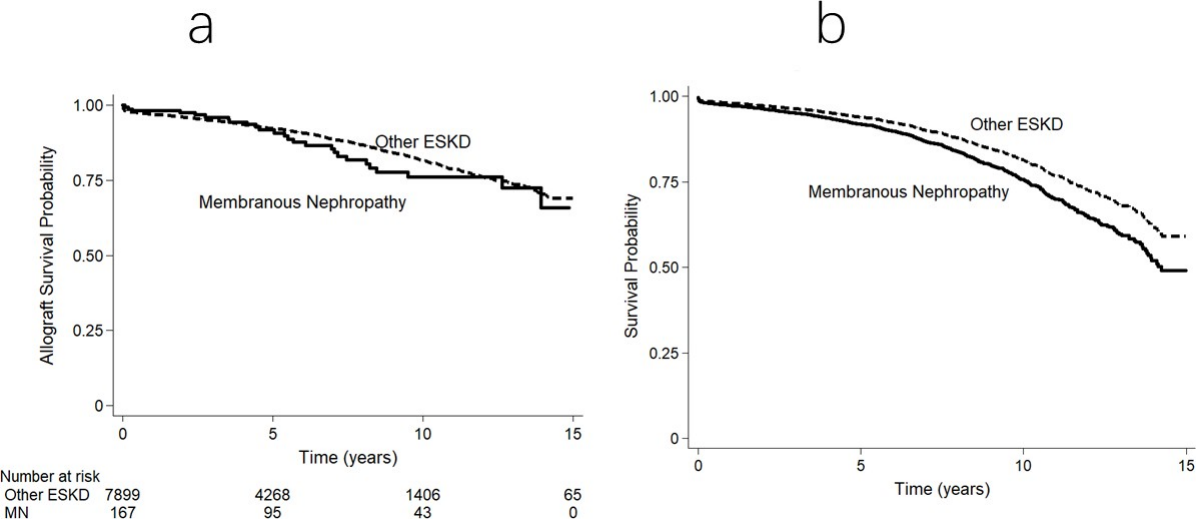
Overall first kidney allograft survival curves for patients undergoing kidney transplantation for ESKD in Australia and New Zealand. (a) Unadjusted Kaplan–Meier survival curve. (b) Survival curve adjusted for demographic, comorbidity and allograft indices. The difference between the groups was not significant (unadjusted p = 0.53; adjusted p = 0.13).

A total of 167 (40%) patients with MN received 172 kidney allografts during the study period. Respective unadjusted first allograft survival rates at 1, 5, and 10-years were 98%, 92% and 76% in patients with MN and 97%, 92% and 82% in patients with other causes of ESKD. The risk of first allograft loss was not significantly associated with MN (aHR 1.35, 95% CI 0.91–2.01, p = 0.13 when compared with all other ESKD, Fig 3; and aHR 1.43, 95% CI 0.95–2.14, p = 0.08 when compared with other forms of GN). Causes of allograft failure were shown in S5 Table. Glomerulonephritis was a more common cause of allograft loss in patients with MN than in those with other causes of ESKD (38% vs. 8%).

Unadjusted death-censored allograft survival rates at 1, 5, and 10-years were 99%, 93% and 79% in patients with MN and 98%, 94% and 87% in patients with other causes of ESKD. The hazard of death-censored kidney allograft loss was higher in patients with MN than in those with other causes of ESKD using either Cox regression analysis (aHR 1.55, 95% CI 1.00–2.41, p = 0.05) (S4 Fig) or competing-risk analysis (SHR 1.59, 95% CI 1.07–2.38, p = 0.02; S5 Fig). Similar results were observed when patients with MN were compared to patients with other forms of GN (aHR 1.57, 95% CI 1.00–2.46, p = 0.05; SHR 1.63, 95% CI 1.09–2.44, p = 0.02).

### Kidney transplant patient survival

**Fig 4:**
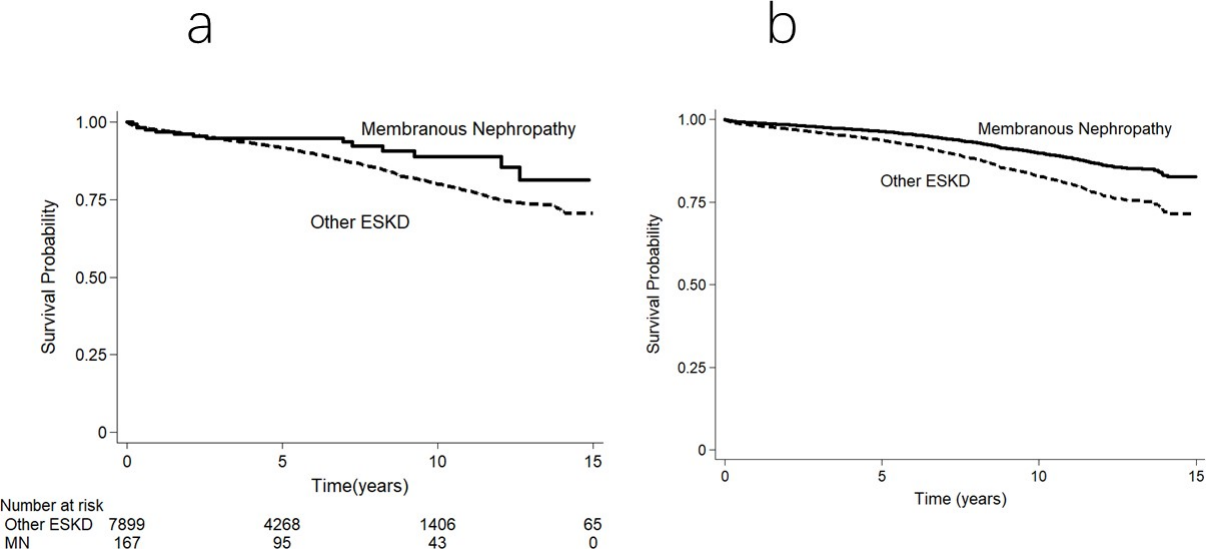
Transplant patient survival curves for individuals undergoing first kidney transplantation for ESKD in Australia and New Zealand. (a) Unadjusted Kaplan–Meier survival curve. (b) Survival curve adjusted for demographic, comorbidity and allograft indices. The difference between the 2 groups was significant in the adjusted analyses (unadjusted p = 0.049; adjusted p = 0.04).

The unadjusted patient survival rates for transplant patients at 1, 5 and 10-years were 97%, 95%, 89% for MN, and 98%, 92%, 80% for other ESKD, respectively. The causes of death were shown in S5 Table. The risk of all-cause mortality was significantly lower in patients with MN (aHR 0.57, 95% CI 0.33–0.97, p = 0.04) (Fig 4). A similar result was observed using competing-risk analysis (SHR 0.55, 95% CI 0.32–0.94, p = 0.03) (S6 Fig).

When patients with MN were compared to patients with other forms of GN, no significant difference in patient survival was observed using either Cox regression analysis (aHR 0.64, 95% CI 0.37–1.11, p = 0.11) or competing-risk analysis (SHR 0.63, 95% CI 0.35–1.10, p = 0.10).

## Discussion

This retrospective, multicenter, two- countries registry analysis demonstrated that MN was independently associated with lower mortality, higher transplantation rates and comparable dialysis-independent kidney function recovery during dialysis treatment compared with other causes of ESKD. Following first kidney transplantation, MN was associated with comparable overall allograft survival, higher recurrence and inferior death-censored allograft survival but better patient survival compared to other ESKD. When only patients with ESKD secondary to glomerulonephritis were taken into consideration, the outcomes of patients with MN were similar to those with other forms of GN, except that MN was associated with higher rates of kidney transplantation, higher primary kidney disease recurrence rates after transplantation and inferior death-censored allograft survival.

Preceding studies of clinical outcomes in patients with MN have primarily focused on earlier stages of chronic kidney disease prior to reaching ESKD [5, 11–14]. The findings of the present study share some similarities with those of a previously published US Renal Data System (USRDS) study of survival outcomes in 84,301 patients with ESKD attributed to glomerulonephritis [8], which observed considerable (>4-fold) variability in overall mortality rates across glomerulonephritis subtypes. The present study similarly demonstrated heterogeneity in clinical outcomes in patients with ESKD according to type of glomerulonephritis, in that patients with MN experienced a higher rate of primary kidney disease recurrence and death-censored allograft loss than patients with other forms of glomerulonephritis. In contrast, no differences were observed for dialysis or transplant patient survival. However, the results of the two studies were not comparable because of the different comparator (non-MN glomerulonephritis versus IgA nephropathy), different survival analyses (separated versus combined dialysis and kidney transplant analyses), and higher amounts of missing data in the USRDS study (0.5% at the utmost vs 32%).

Interestingly, 3% of patients commencing dialysis for ESKD secondary to MN experienced kidney recovery and were able to stop dialysis. Whilst the frequency of this event was not significantly different to that experienced by patients with other causes of ESKD or other forms of glomerulonephritis, the findings do support those of the previous ANZDATA registry analyses [15, 16] and highlight that caution should be exercised when considering early kidney transplantation in ESKD patients. Previous ANZDATA studies reported the common reasons [15,16] for recovery were autoimmune renal disease, hemolytic-uremic syndrome, paraproteinemia, cortical necrosis, renovascular disease, and obstructive uropathy. Macdonald [16] reported recovery was significantly more likely in patients with higher baseline eGFR, and with no hypertension or peripheral vascular disease.

Nonetheless, patients with ESKD due to MN were significantly more likely to receive kidney transplantation compared to patients with other ESKD or other glomerulonephritis. This finding was perhaps not altogether surprising given that patients with MN were less likely to be referred late to a renal service and demonstrated less comorbidity burden. However, even after adjustment for these variables, MN patients were still significantly more likely to undergo kidney transplantation, suggesting some residual confounding factors. These results contrast with the findings of a USRDS study in which patients with MN were significantly less likely to receive transplantation compared to those with IgA nephropathy (HR 0.88, 95% CI 0.83–0.93) [17]. However, these data cannot be directly compared due to different reference groups (all other GN/ESKD vs. IgA nephropathy).

Following kidney transplantation, patients with MN experienced worse death-censored graft survival rates compared to patients with other causes of ESKD or patients with other forms of glomerulonephritis. Similar results were reported by Moroni et al [7] in which death-censored kidney allograft survival rates were lower in 35 first kidney transplant patients with MN in a single Italian center compared with 70 contemporary transplant controls (graft survival at 5-year: 82% vs 90%, at 10-year: 58% vs 69%). Although the result was not quite statistically significant (p = 0.06), the statistical power was limited by the relatively smaller sample size (n = 35) compared to that of the present study (n = 167). Using data from the UK Renal Registry, Pruthi et al also demonstrated higher kidney allograft failure rates in kidney transplant recipients with MN compared with those with autosomal dominant polycystic kidney disease (ADPKD, aHR 2.0, 95% CI 1.4–2.9) [18]. Similar results regarding the higher death-censored allograft failure risk were reported for patients with MN in the analysis of transplant patients with GN in a European Renal Association-European Dialysis and Transplant Association Registry study (n = 14383, aHR 1.65, 95% CI 1.40–1.95 at 15-year) compared with ADPKD [19] and a USRDS study [20] (n = 32,131, HR 1.27, 95% CI 1.14 to 1.41) compared with IgA nephropathy.

A significant factor underpinning the reduced allograft survival rate in patients with MN was the relatively frequent recurrence of MN in the kidney allograft. In the present study, the biopsy-proven recurrence rate of MN after kidney transplantation was 11.4% after a median time period of 3.6 years, which was at the lower end of the range in the literature (7%-44%) [3, 7, 21–27]. The variability in reported rates of recurrence was primarily related to difference in follow-up durations and indications for performing a kidney transplant biopsy (clinical only versus routine surveillance protocol) [21, 27–29]. Estimation of the rate of recurrence of MN in kidney allografts was further complicated by the fact that de novo MN can occur in kidney allografts with a reported incidence of 0.7–9.3% [26, 27, 30–33]. Of those patients who experienced MN recurrence in the present study, 47% progressed to allograft failure after a median period of 3.0 years from the time of diagnosis of recurrence. Again, this was similar to what has been reported in the literatures (40%-69%) [5, 21, 24–26, 34, 35].

Despite poorer death-censored graft survival, overall patient survival in patients with MN was superior to that of patients with other causes of ESKD and similar to that of patients with other forms of glomerulonephritis, such that overall allograft failure was comparable amongst these groups. Similar graft survival between MN and other GN were found in a previous smaller ANZDATA Registry study involving 81 transplant patients with MN (unadjusted HR 1.58, 95% CI: 0.71–3.50) [24] and a Taiwan study of 85 post-transplantation glomerulonephritis including eight cases with MN (p = 0.46)[25]. With regard to patient survival after transplantation, the results in a few studies [5, 17, 18, 33], all with small patient numbers and different comparator group, have generally been comparable. Similar patient survivals for MN were found in a UK registry study involving 183 patients with MN compared to ADPKD (aHR 0.91, 95% CI 0.61–1.36) [18] and a study by Moroni et al of 35 patients with MN compared to other ESKD (96% vs 88% at 15-year, p = 0.6) [7]. Sprangers et al reported that recurrent MN (n = 15) was not a predictor of patient survival [22]. However, higher mortality of MN after transplantation was observed in a study using USRDS data with 2,249 patients with MN compared with IgA nephropathy (HR 1.52, 95% CI 1.34–1.72) [20]. The apparent lack of adverse impact of MN on patient survival despite poorer death-censored kidney allograft survival in our study might be related to improvements in immunosuppressive therapy. The anti-PLA2R antibody in the serum is unlikely to have had an impact as the study period was prior to the introduction of this antibody in clinical practice [22, 36–38]. Patients with MN also had a number of favorable characteristics for survival, including greater frequency of early renal service referral and a higher likelihood of pre-emptive kidney transplantation. Furthermore, compared to patients with ESKD from other causes, MN patients exhibited fewer comorbidities. Although these characteristics had been adjusted for in the statistical models, the possibility of residual confounding remained.

The strengths of this study included its large sample size and inclusiveness. These strengths should be balanced against the study’s limitations, which included heterogeneity in ESKD care and limited depth of collected information. Even though adjustment was made for a number of demographic, clinical and RRT variables, residual confounding was possible. The ANZDATA Registry does not collect information on the reasons for recovery of renal function, presence of nephrotic syndrome, anti-PLA2R antibody measurements or immunosuppressive treatment of post-transplant recurrence. Some patients with MN may have had secondary MN. The frequency of MN recurrence in kidney allografts may have been under-estimated as not all MN patients who experienced kidney allograft failure may have received a kidney biopsy.

In conclusion, MN was associated with superior survival on dialysis and following kidney transplantation compared to patients with other causes of ESKD, but higher rates of death-censored allograft loss as a result of MN recurrence. Compared to patients with other forms of glomerulonephritis, patients with MN have comparable dialysis and kidney transplant outcomes, except for higher death-censored graft failure and disease recurrence rates. These findings should be used to better inform shared decision-making by patients with MN and clinicians regarding ESKD care.

## Supporting information

S1 Table Characteristics of patients with ESKD due to glomerulonephritis (GN) whose first RRT was dialysis in Australia and New Zealand. **Abbreviations:** ESKD, End-stage kidney diseases; RRT, Renal replacement therapy; ATSI, Aboriginal and Torres Strait Islander; MPI, Maori and Pacific Islander; BMI, body mass index.
(DOC)

S2 Table Causes of death of ESKD patients whose first RRT was dialysis in Australia and New Zealand. **Abbreviations:** ESKD, End-stage kidney disease; RRT, Renal replacement therapy.
(DOC)

S3 Table Characteristics of patients with ESKD due to glomerulonephritis (GN) who received their first kidney allograft in Australia and New Zealand. ^a^ Fisher’s test result. **Abbreviations:** ESKD, End-stage kidney diseases; MN, Membranous nephropathy; ATSI, Aboriginal and Torres Strait Islander; MPI, Maori and Pacific Islander; RRT, Renal replacement therapy; BMI, body mass index; EBV, Epstein-Barr Virus; CMV, Cytomegalovirus.
(DOC)

S4 Table Recurrence of primary kidney disease and attendant clinical outcomes of kidney transplant recipients in Australia and New Zealand. **Abbreviations:** MN, Membranous nephropathy; ESKD, End-stage kidney disease; GN, Glomerulonephritis.
(DOC)

S5 Table Causes of death and kidney allograft failure in patients undergoing first kidney transplantation for ESKD in Australia and New Zealand. ^a^ Fisher’s test result. ^b^ For difference of chronic rejection between patients with ESKD secondary to membranous nephropathy and other ESKD. **Abbreviations:** ESKD, End-stage kidney disease
(DOC)

S1 Fig Cumulative probability of dialysis-independent recovery of kidney function in patients commencing dialysis for ESKD in Australia and New Zealand. (a) Unadjusted curve. (b) Adjusted curve by demographic and comorbidity indices. The difference between the 2 groups were not significant (unadjusted p = 0.20; adjusted p = 0.19).
(TIF)

S2 Fig Cumulative incidence function(CIF) curves by competing- risk analysis of the dialysis-independent kidney function recovery for ESKD patients in Australia and New Zealand. The difference between the 2 groups was not significant (p = 0.14).
(TIF)

S3 Fig Cumulative hazard curve of undergoing kidney transplantation for patients receiving dialysis for ESKD in Australia and New Zealand. (a) Unadjusted curve. (b) Adjusted curve by demographic and comorbidity indices. The difference between the 2 groups was significant (unadjusted p < 0.001; adjusted p < 0.001)
(TIF)

S4 Fig Death-censored first kidney allograft survival curves for patients undergoing kidney transplantation for ESKD in Australia and New Zealand. (a) Kaplan–Meier survival curve. (b) Survival curve adjusted for demographic, comorbidity and allograft indices. The difference between the 2 groups was not significant (unadjusted p = 0.28; adjusted p = 0.05).
(TIF)

S5 Fig Cumulative incidence function (CIF) curves by competing risk analysis of allograft failure for patients undergoing first kidney transplantation for ESKD in Australia and New Zealand. The difference between the 2 groups was significant (p = 0.02).
(TIF)

S6 Fig Cumulative incidence function (CIF) curves by competing risk analysis of mortality for patients undergoing first kidney transplantation for ESKD in Australia and New Zealand. The difference between the 2 groups was significant (p = 0.03).
(TIF)

S1 File STROBE checklist. (DOCX)

S2 File PLOS ONE clinical studies checklist. (DOCX)

S3 File (DTA)
