# Supplementary material for: Long-term outcomes of patients with end-stage kidney disease due to membranous nephropathy: A cohort study using the Australia and New Zealand Dialysis and Transplant Registry
Source: PLoS One. 2019 Aug 23;14(8):e0221531. doi: 10.1371/journal.pone.0221531 (PMC6707602; doi:10.1371/journal.pone.0221531)
Supplement: S1 Table — Abbreviations: ESKD, End-stage kidney diseases; RRT, Renal replacement therapy; ATSI, Aboriginal and Torres Strait Islander; MPI, Maori and Pacific Islander; BMI, body mass index. (DOC) [file pone.0221531.s001.doc]

**S1 Table.**

| **Characteristics** | **Membranous Nephropathy (n=388)** | **Other GN**  **(n=7,409)** | **P value** |
| --- | --- | --- | --- |
| **Age (years)** | 61[51-70] | 55[41-68] | <0.001 |
| 18-29 | 12 (3%) | 699(9%) | <0.001 |
| 30-39 | 27 (7%) | 893(12%) |  |
| 40 -49  50-59 | 45 (12%)  98 (25%) | 1,327(18%)  1,398(19%) |  |
| 60-69 | 105 (27%) | 1,515(21%) |  |
| >70 | 101 (26%) | 1,577(21%) |  |
| **Male** | 288 (74%) | 4,682 (63%) | <0.001 |
| **Racial origin** |  |  | <0.001 |
| Caucasian | 337 (87%) | 5,592 (75%) |  |
| ATSI | 11(3%) | 407 (6%) |  |
| MPI | 19 (5%) | 525 (7%) |  |
| Asian | 17 (4%) | 688 (9%) |  |
| Other | 4 (1%) | 197 (3%) |  |
| **RRT Era** |  |  | 0.85 |
| 1998 – 2002 | 142 (37%) | 2,802 (38%) |  |
| 2003 – 2007 | 156 (40%) | 2,879 (39%) |  |
| 2008 -2012 | 90 (23%) | 1,728 (23%) |  |
| **Smoking status at RRT entry** |  |  | 0.02 |
| Current | 60(15%) | 1,068 (14%) |  |
| Former | 170 (44%) | 2,797 (38%) |  |
| Never | 158 (41%) | 3,537 (48%) |  |
| **Diabetes mellitus** | 63 (16%) | 946 (13%) | 0.05 |
| **Chronic lung disease** | 70 (18%) | 1,038 (14%) | 0.03 |
| **Coronary artery disease** | 134 (35%) | 1,829 (25%) | <0.001 |
| **Peripheral vascular disease** | 67 (17%) | 832 (11%) | <0.001 |
| **Cerebrovascular disease** | 48 (12%) | 641 (9%) | 0.01 |
| **BMI (kg/m^2^)** | 26[23-29] | 26[23-30] | 0.63 |
| Underweight (<18.5) | 13(3%) | 280(4%) | 0.69 |
| Normal weight (18.5-24) | 152(39%) | 2,974(40%) |  |
| Overweight (25-29) | 136(35%) | 2,381(32%) |  |
| Obese (>30) | 87(23%) | 1,754(24%) |  |
| **Late referral** | 70 (18%) | 1,794 (24%) | 0.01 |
| **First RRT** |  |  | 0.05 |
| Hemodialysis | 271 (70%) | 5,509 (74%) |  |
| Peritoneal dialysis | 117 (30%) | 1,900 (26%) |  |
| **Follow-up years** | 3.3[1.6– 5.6] | 3.4[1.5–5.6] | 0.70 |
| **Native kidney biopsy** | 388(100%) | 5,487(74%) | <0.001 |
