# Supplementary material for: Long-term outcomes of patients with end-stage kidney disease due to membranous nephropathy: A cohort study using the Australia and New Zealand Dialysis and Transplant Registry
Source: PLoS One. 2019 Aug 23;14(8):e0221531. doi: 10.1371/journal.pone.0221531 (PMC6707602; doi:10.1371/journal.pone.0221531)
Supplement: S2 Table — Abbreviations: ESKD, End-stage kidney disease; RRT, Renal replacement therapy. (DOC) [file pone.0221531.s002.doc]

**S2 Table.**

| **Characteristics** | **Membranous Nephropathy**  **(n=169)** | **Other ESKD**  **(n=17,947)** | **P value** |
| --- | --- | --- | --- |
| **Death** |  |  | 0.23 |
| Cardiac | 71(42%) | 7,547(42%) |  |
| Vascular | 24(14%) | 2,670(15%) |  |
| Fatal Infection | 10(6%) | 2,061(11%) |  |
| Dialysis Withdrawal | 32(19%) | 2,804(16%) |  |
| Malignancy | 18(11%) | 1,727(10%) |  |
| Other | 14(8%) | 1,138(6%) |  |
