# Supplementary material for: Long-term outcomes of patients with end-stage kidney disease due to membranous nephropathy: A cohort study using the Australia and New Zealand Dialysis and Transplant Registry
Source: PLoS One. 2019 Aug 23;14(8):e0221531. doi: 10.1371/journal.pone.0221531 (PMC6707602; doi:10.1371/journal.pone.0221531)
Supplement: S3 Table — a Fisher’s test result. Abbreviations: ESKD, End-stage kidney diseases; MN, Membranous nephropathy; ATSI, Aboriginal and Torres Strait Islander; MPI, Maori and Pacific Islander; RRT, Renal replacement therapy; BMI, body mass index; EBV, Epstein-Barr Virus; CMV, Cytomegalovirus. (DOC) [file pone.0221531.s003.doc]

**S3 Table.**

| **Characteristics** | **MN**  **(n=167)** | | **Other GN**  **(n=3,411)** | **P value** |
| --- | --- | --- | --- | --- |
| **Recipient Age** (years) | 53[40-61] | 47[36-56] | | <0.001 |
| 18-29 | 13(8%) | 437(13%) | | 0.001^a^ |
| 30-39 | 26(16%) | 650 (19%) | |  |
| 40-49 | 34(20%) | 878(26%) | |  |
| 50-59 | 42(25%) | 833(24%) | |  |
| 60-69 | 45(27%) | 553(16%) | |  |
| >70 | 7(4%) | 60 (2%) | |  |
| **Male Recipient** | 129(77%) | 2,232(65%) | | 0.002 |
| **Recipient Race** |  |  | | 0.07^a^ |
| White | 143(86%) | 2,642(77%) | |  |
| Asian | 10(6%) | 423(12%) | |  |
| ATSI | 5(3%) | 90(3%) | |  |
| MPI | 6(4%) | 138(4%) | |  |
| Other | 3(2%) | 118(3%) | |  |
| **Transplant era** |  |  | | 0.003 |
| 1998-2002 | 51(31%) | 688(20%) | |  |
| 2003-2007 | 45(27%) | 1,209(36%) | |  |
| 2008-2013 | 71(42%) | 1,514(44%) | |  |
| **Smoking status at RRT entry** |  |  | | 0.31 |
| Current | 15(9%) | 396(12%) | |  |
| Former | 62(37%) | 1,095(32%) | |  |
| Never | 90(54%) | 1,916(56%) | |  |
| **Diabetes mellitus** | 9(5%) | 159(5%) | | 0.67 |
| **Chronic lung disease** | 16(10%) | 169(5%) | | 0.01 |
| **Coronary artery disease** | 25(15%) | 251(7%) | | <0.001 |
| **Peripheral vascular disease** | 9(5%) | 78(2%) | | 0.02^a^ |
| **Cerebrovascular disease** | 8(5%) | 72(2%) | | 0.03^a^ |
| **BMI (kg/m^2^)** | 25.9[22.8-28.6] | 25.3[22.4-29.0] | | 0.51 |
| Underweight (<18.5) | 5(3%) | 124(4%) | | 0.65 |
| Normal weight (18.5-24) | 69(41%) | 1,483(43%) | |  |
| Overweight (25-29) | 64(38%) | 1,143(34%) | |  |
| Obese (>30) | 29(17%) | 645(19%) | |  |
| **Late referral** | 25(15%) | 642(19%) | | 0.21 |
| **First RRT** |  |  | | 0.01 |
| Kidney transplantation | 29(17%) | 390(11%) | |  |
| Peritoneal dialysis | 46(28%) | 777(23%) | |  |
| Hemodialysis | 92(55%) | 2,244(66%) | |  |
| **Positive recipient** **EBV serology status** | 139(89%) | 2,828(89%) | | 0.83 |
| **Positive recipient CMV serology status** | 115(71%) | 2,263(68%) | | 0.47 |
| **Male donor** | 84(51%) | 1,701(51%) | | 0.98 |
| Male donor for female receipt | 18(11%) | 618(18%) | | 0.02 |
| **Deceased donor** | 96(57%) | 1,922(56%) | | 0.81 |
| **Subsequent allografts** | 5(3%) | 112(3%) | | 1.00^a^ |
| 2^nd^ | 5(100%) | 110(98%) | |  |
| 3^rd^ or more | 0 | 2(2%) | |  |
| **Follow-up years** | 5.6[3.2-10.6] | 5.7[3.1-9.0] | | 0.22 |
| **Native kidney biopsy** | 167(100%) | 2,841(83%) | | <0.001 |
