# Supplementary material for: Long-term outcomes of patients with end-stage kidney disease due to membranous nephropathy: A cohort study using the Australia and New Zealand Dialysis and Transplant Registry
Source: PLoS One. 2019 Aug 23;14(8):e0221531. doi: 10.1371/journal.pone.0221531 (PMC6707602; doi:10.1371/journal.pone.0221531)
Supplement: S4 Table — Abbreviations: MN, Membranous nephropathy; ESKD, End-stage kidney disease; GN, Glomerulonephritis. (DOC) [file pone.0221531.s004.doc]

**S4 Table.**

|  | **Recurrence ratio**  **n(%)** | **Allograft failure after recurrence**  **n(%)** | **Death-censored**  **allograft failure after recurrence**  **n(%)** | **Death after**  **Recurrence**  **n(%)** |
| --- | --- | --- | --- | --- |
| **Total** | 190(2.4) | 93(49) | 72(37) | 27(14) |
| **MN**  (n=167) | 19(11.4) | 9(47) | 7(37) | 2(11) |
| **Other ESKD**  (n=7,899) | 171(2.2) | 84(49) | 65(38) | 25(15) |
| **Other GN**(n= 3,411) | 152(4.5) | 74(49) | 59(39) | 19(13) |
