# Supplementary material for: Long-term outcomes of patients with end-stage kidney disease due to membranous nephropathy: A cohort study using the Australia and New Zealand Dialysis and Transplant Registry
Source: PLoS One. 2019 Aug 23;14(8):e0221531. doi: 10.1371/journal.pone.0221531 (PMC6707602; doi:10.1371/journal.pone.0221531)
Supplement: S5 Table — a Fisher’s test result. b For difference of chronic rejection between patients with ESKD secondary to membranous nephropathy and other ESKD. Abbreviations: ESKD, End-stage kidney disease (DOC) [file pone.0221531.s005.doc]

**S5 Table.**

| **Characteristics** | **Membranous nephropathy (n=167)** | **Other**  **ESKD**  **(n=7,899)** | **P value** |
| --- | --- | --- | --- |
| **Death** | 14(8%) | 975(12%) | 0.12 |
| Cardiac | 3(21.5%) | 264(27%) | 0.047^a^ |
| Vascular | 0(0%) | 106(11%) |  |
| Fatal Infection | 2(14%) | 206(21%) |  |
| Dialysis Withdrawal | 3(21.5%) | 32(3%) |  |
| Malignancy | 5(36%) | 238(25%) |  |
| Other | 1 (7%) | 129(13%) |  |
| **Overall allograft failure** | 26(16%) | 926(12%) | 0.13 |
| Acute rejection | 3(12%) | 62(7%) | < 0.001^a^ |
| Chronic rejection | 11(42%) | 456(49%) | 0.49^b^ |
| Thrombosis | 0(0) | 90(10%) |  |
| Glomerulonephritis | 10(38%) | 72(8%) |  |
| Other | 2(8%) | 246(26%) |  |
