# Supplementary figures and images for: Long-term outcomes of patients with end-stage kidney disease due to membranous nephropathy: A cohort study using the Australia and New Zealand Dialysis and Transplant Registry
Source: PLoS One. 2019 Aug 23;14(8):e0221531. doi: 10.1371/journal.pone.0221531 (PMC6707602; doi:10.1371/journal.pone.0221531)

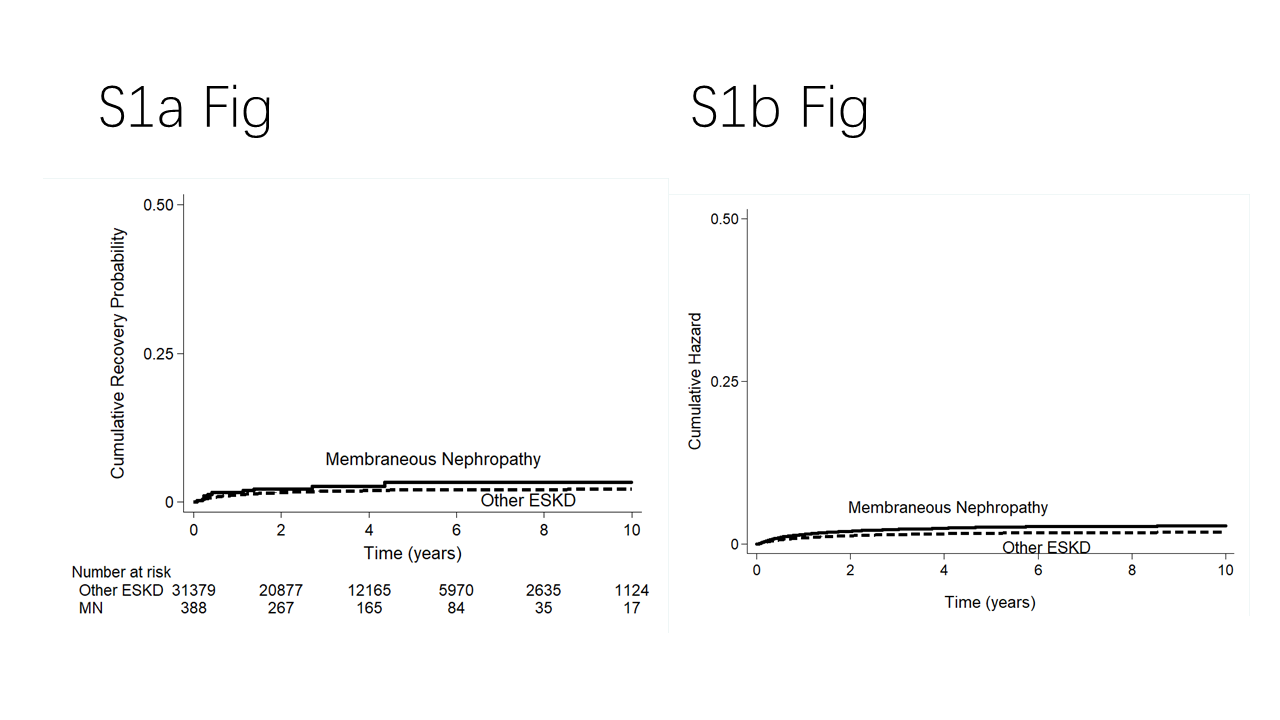

Supplement: S1 Fig — (a) Unadjusted curve. (b) Adjusted curve by demographic and comorbidity indices. The difference between the 2 groups were not significant (unadjusted p = 0.20; adjusted p = 0.19). (TIF) [file pone.0221531.s006.tif]

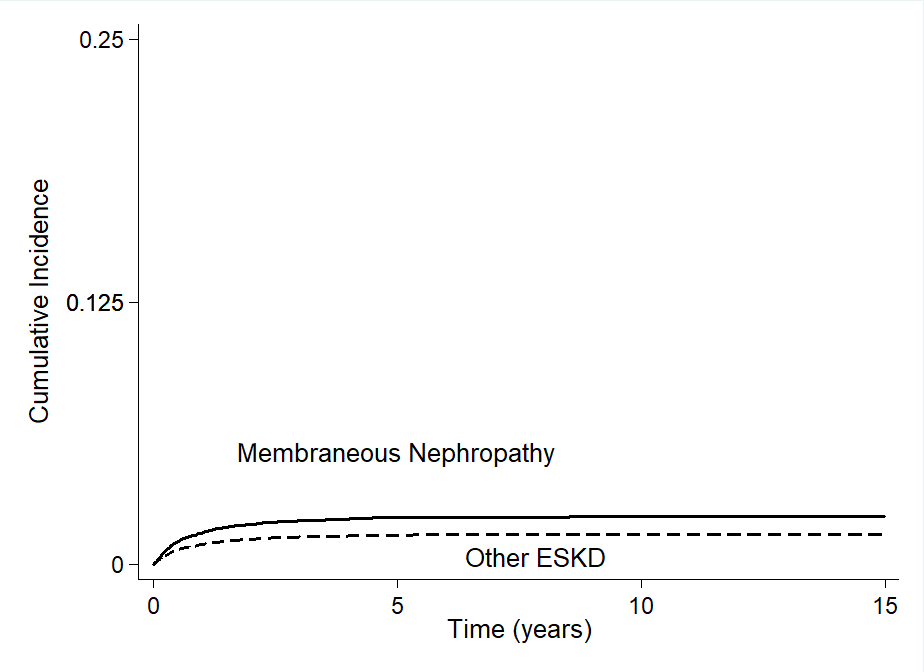

Supplement: S2 Fig — The difference between the 2 groups was not significant (p = 0.14). (TIF) [file pone.0221531.s007.tif]

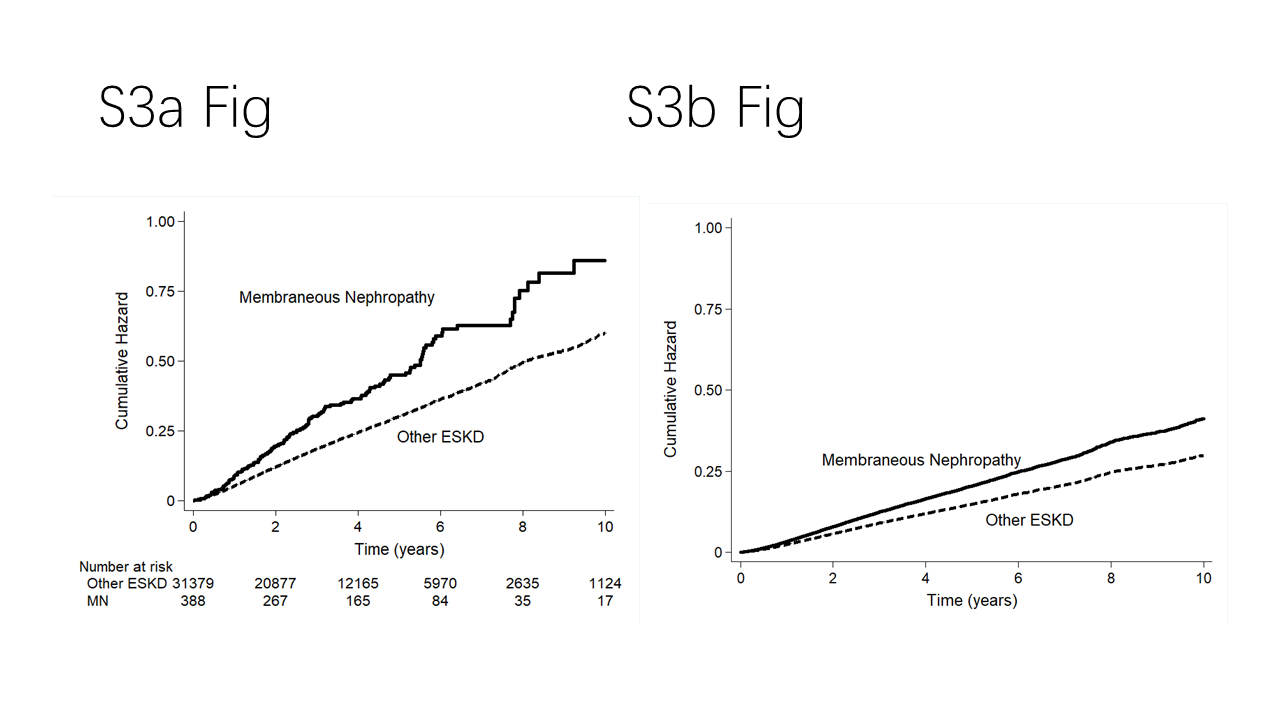

Supplement: S3 Fig — (a) Unadjusted curve. (b) Adjusted curve by demographic and comorbidity indices. The difference between the 2 groups was significant (unadjusted p < 0.001; adjusted p < 0.001) (TIF) [file pone.0221531.s008.tif]

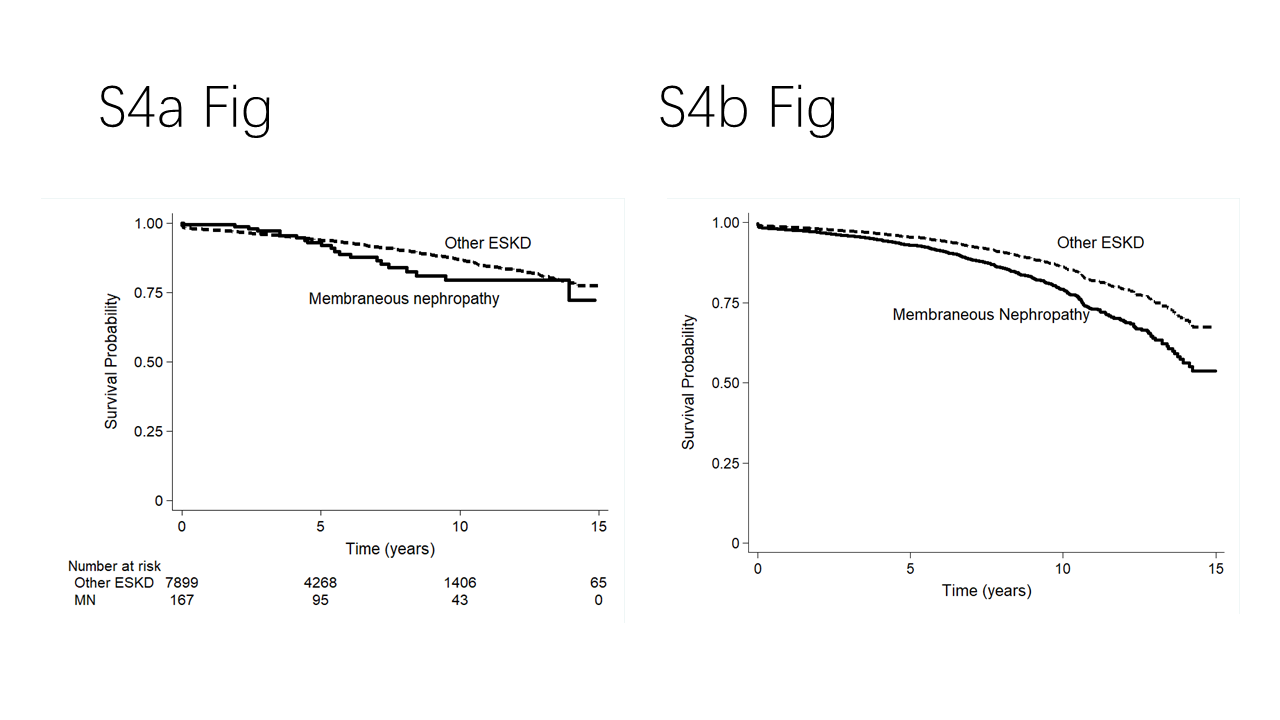

Supplement: S4 Fig — (a) Kaplan–Meier survival curve. (b) Survival curve adjusted for demographic, comorbidity and allograft indices. The difference between the 2 groups was not significant (unadjusted p = 0.28; adjusted p = 0.05). (TIF) [file pone.0221531.s009.tif]

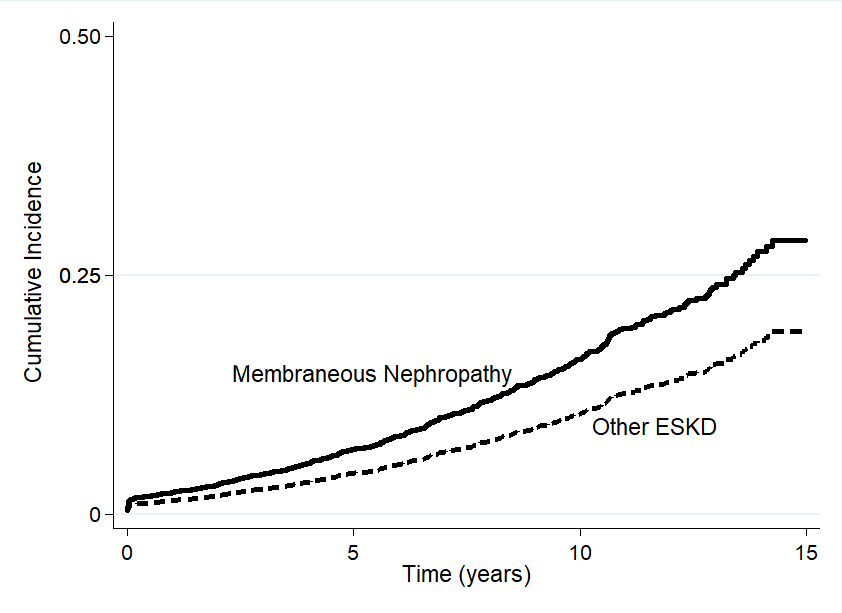

Supplement: S5 Fig — The difference between the 2 groups was significant (p = 0.02). (TIF) [file pone.0221531.s010.tif]

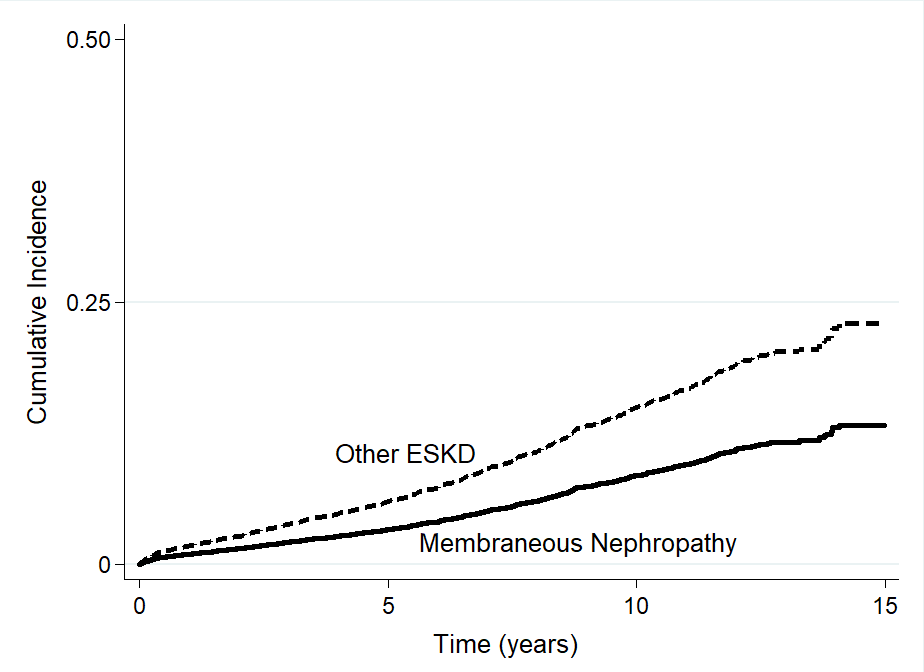

Supplement: S6 Fig — The difference between the 2 groups was significant (p = 0.03). (TIF) [file pone.0221531.s011.tif]
